# The assessment of osteoporosis risk factors in Iranian women compared with Indian women

**DOI:** 10.1186/1471-2474-9-28

**Published:** 2008-02-27

**Authors:** Afsaneh Keramat, Bhushan Patwardhan, Bagher Larijani, Arvind Chopra, Ambrish Mithal, Devlina Chakravarty, Hossein Adibi, Ahmad Khosravi

**Affiliations:** 1 Shahroud University of Medical Sciences, Hafte Tir Avenue, Shahroud, Iran; 2 School of Health Sciences, University of Pune, India; 3 Center of Rheumatoid Diseases, Pune, India; 4 Endocrinology and Metabolism Research center, Tehran University of Medical Sciences, Iran; 5 Endocrinology Department of Appolo Hospital, New Delhi, India; 6 Radiology Department of Max Medical Center, New Delhi, India

## Abstract

**Background:**

Osteoporosis is an important public health problem in older adults. It is more common in postmenopausal women and not only gives rise to morbidity but also markedly diminishes the quality of life in this population. There is lack of information about the risk factor of osteoporosis in developing countries. In this study we aimed to assess the risk factors for osteoporosis in postmenopausal women from selected BMD centers of two developing Asian countries (Iran and India).

**Methods:**

This study is a multicenter interview-based study conducted in selected hospitals and health centers from urban areas in Iran and India. The case group included postmenopausal osteoporotic women who were identified as patients with bone density higher than 2.5 SD below average of young normal bone density (in L1–L4) spine region interest and/or total femoral region) by using DEXA method. The controls were chosen from postmenopausal women with normal bone density (in L1–L4 spine and total femoral regions using DEXA method) matching in age groups was strategy of choice.

The sample sizes included from Iran a total of 363 subjects (178 osteoporotic and 185 normal) and from India a total of 354 subjects (203 osteoporotic and 151 normal).

**Results:**

The significant (p < 0.05) risk factors in present study population with their Odds Ratios (in parenthesis, respectively in Iran and India) were as follow:

Lower education defined as less than class 12 or nil college (2.1) (2.7), duration of menopause greater than 5 years: (2.2) (1.4), Menarche age (after 14 years): (1.9) (1.6), Menopause age (before 45 years): (1.1) (2), Parity more than 3: (1.1) (1), Bone and joint problem (2.3) (2.2).

Calcium supplementation (0.6) and HRT (0.4) were shown as protective factors and steroid therapy (3.3) was found as a risk factor in Iran. Calcium supplementation more than 1 year (0.3) was shown as a protective factor in India.

Pure vegetarianism: (2.2) and Red meat consumption more than 4 times per week (1.4) was shown as a risk factor in Indian and Iranian subjects respectively.

Regular consumption of Soya (0.3), almond (0.5), fish (0.5), fruits (0.4) and milk tea 4 cups per day and more (0.4) appeared to be significant protective factors in India. Regular consumption of cheese (0.5), milk (0.5), chicken (0.4), egg (0.6), fruit (0.4), tea 7 cups per day and more (0.3) were found to be significant protective factors in Iran. Exercises were shown as protective factor in Iran (0.4) and India (0.4).

There were no significant differences in association of risk factors and osteoporosis between Iranian and Indian subjects.

**Conclusion:**

Osteoporosis in Iranian and Indian subjects also appears to be associated with several known risk factors that well described in the literature. There were no significant differences in association of risk factors and osteoporosis between Iranian and Indian subjects. It was shown a protective role of certain nutritional dietary components and also exercises in both populations and can be exploited in preventive educational strategies on osteoporosis in these populations.

## Background

Osteoporosis and low bone density are significant risk factors for morbidity and mortality in older adults. These conditions are characterized by poor bone strength and are associated with an increased risk of the fractures from even slightly traumatic events. Several medications have recently been labeled for the treatment of osteoporosis, but their marginal benefits require careful consideration of their cost. Prevention is preferable to treatment since no therapy fully restores lost bone mass. It is also known that the prevalence of osteoporosis varies from country to country, and within countries [1]. Differences in race, nutritional status, physical activity, lifestyle and living conditions all contribute to its variability [2]. Several demographic factors may be considered as barriers to health prevention like high rate of illiteracy and low socioeconomic status in developing countries [3]. Other factors that may contribute to regional difference include water hardness, sunlight exposure, poverty levels and the proportional agricultural land. Further studies are needed to identify the environmental factors responsible for such marked regional difference [4].

Osteoporosis is an important public health problem in older adults. Not only does it give rise to morbidity but also markedly diminishes the quality of life of women after menopause, and of both women and men over 65 years of age [5]. In the twentieth century the proportion of older persons started to rise and is expected to continue throughout this century. The number of individuals aged 60 and above is projected to grow to almost 2 billion by 2050, of who fifty-four percent live in Asia and the vast majority of who will be in the developing world. Such accelerated global population aging will increase economic and social demands on all countries [6].

On the other hand the estimated lifetime risk of osteoporotic fracture is as high as 50 percent, especially in white and Asian women. At present in India osteoporotic fractures usually occur 10 to 20 years earlier in men and women compared to Caucasian in the west. In Iran the prevalence of lumbar spine osteoporosis and osteopaenia among post-menopausal women was reported 26.7 and 50%, respectively [7].

The attainment of a higher peak bone density has an important role in the prevention of osteoporosis later in life [8-11]. Genetic factors and race/ethnicity have a strong influence on peak bone density [11-14]. Physiological, environmental and modifiable lifestyle factors can also play a significant role [13,15]. These factors include adequate nutrition and body weight [16,17], exposure to sex hormones at puberty [18,19] and level of physical activity [20]. They are not only important for the acquisition of maximal bone mass but also for its maintenance throughout life [13,21-23].

Definition and comparison of osteoporosis risk factors in these two developing Asian countries (Iran and India) that have some similarities and differences in culture and life style can be useful for preventive health programs of Osteoporosis. In this study the risk factors of osteoporosis were compared in postmenopausal women whose bone mineral density (BMD) were measured in selected BMD centers of Iran and India during May 2002 to January 2005.

## Methods

This study is a case-control, multicentral-based study conducted in selected hospitals and health centers from urban areas in Iran and India. *The case group *included postmenopausal Osteoporotic women who were identified as patients with bone density higher than 2.5 SD below average of young normal bone density (in L1–L4) spine region interest and/or total femoral region) by using DEXA method. The *Controls *were chosen from post-menopausal women with normal bone density (BMD lesser than 1 SD below average of young's in L1–L4 spine and total femoral regions using DEXA method) frequency of matching in age groups was the strategy of choice.

At present study all information about BMDs comes from DEXA method mostly using Lunar machines (in Iran Lunar DPX and in India mostly Lunar DPX and Lunar Prodigy)

There was little information that came from using Hologic Delphi DEXA machine in India. This limitation does not affect on the study results because following solutions controlled it: 1) BMDs did not compare with each other directly and subject divided in two osteoporotic and control (with normal BMD) groups in based of WHO definition. 2) Excluding subjects with osteopenic bone density and making gap between cases' and controls' BMDs (there was no chance for any overlap between cases and controls even by using different BMD machines).

The sample was selected from all postmenopausal women whose bone mineral density was measured in selected centers during the study period (2002 to 2005). Among this population all the osteoporotic women were recalled and interviewed upon their consent to participate as case groups. Controls were selected by recalling candidates from a list of non-osteoporotic non-osteopenic women created from the centers' databases during the study period. Of women contacted for the study, 363 subjects from Iran (178 osteoporotic and 185 normal) and 354 subjects (203 osteoporotic and 151 normal) agreed to participate. Consent to participate was verbal for all of the samples from the centers in Iran and India except KEM hospital that was written because of the rules of hospital.

For control the effect of age on the study results we have tried to match the controls with cases in 10 years groups (table 1) but this kind of matching could not control the effect of age completely thus we have adjusted the result for age for tight control the effect.

**Table 1:** Characteristics of subjects in two populations

	Iran			India		
	
	Case (n = 178)	Control (n = 185)	p-value	Case (n = 203)	Control (n = 151)	p-value
Age(SD)	58.2(7.1)	55.7(6.0)	<0.05	58.9(8.1)	56.4(7.5)	<0.05
Age group (%)						
<50	16(9.0)	15(8.2)		18(9.0)	19(12.8)	
50–59	100(56.2)	124(67.4)	NS	103(51.2)	87(58.8)	NS^1^
60–69	55(30.9)	40(21.7)		66(32.8)	36(24.3)	
>69	7(3.9)	5(2.7)		14(7.0)	6(4.1)	
Anthropometric characters (SD)						
Weight (Kg)	64.5(10.6)	71.9(10.0)	<0.05	60.5(12.8)	71.1(11.1)	<0.05
Height (Cm)	155.8(6.6)	157.1(5.6)	<0.05	152.8(7.5)	157.5(6.4)	<0.05
BMI	26.7(4.4)	29.2(4.5)	<0.05	26.1(5.6)	29.0(4.2)	<0.05

1 = Non significant

Data collected for this study included filling questionnaires through personal interviews, use of case records, files and documents. The questionnaire covered the following factors and information:

- Demographic characteristics including age, education, occupation, marital status

- Menstrual and obstetrical history: menarche age, age of menopause, parity and abortion.

- Medical condition and medication. Medical condition included (history of endocrine disorders like diabetes and thyroid, heart disease, kidney, asthma, and other related medical problem) and also bone and joint disorders (in this study included any disorders and discomfort related to bone and joint that needs treatment and or rest)

Medication included most common related drugs and supplements like: calcium supplementation, HRT and steroids with at least lowest available therapeutic and/or preventive dose that were used continuously 6 months or more for calcium and HRT and one month or more for steroids.

- Nutrition questionnaire: life time food frequency questionnaire and food habits.

- Physical activity, exercises, self-imagination, reporting physical activity and standing on feet.

- Habits: alcohol consumption, smoking, tobacco use, misheri

-Anthropometric characters: height, weight, BMI (weight and height were used to be measured and recorded in all BMD centers before measurement of bone density routinely)

Statistical analysis was performed using SPSS software. We have used Odds Ratio for estimation the association of risk factors with osteoporosis, Multinominal logistic regression was the main method for adjustment of confounds variables. Χ^2 ^was carried out to assess the nominal variables and T student test were used for comparing means of quantitative variables. The 5% level of statistical significance was chosen.

### Ethical issues

Ethic committee clearance and permission obtained whenever based on requirement of participation center. This study was approved by Ethics Committee of Endocrinology and Metabolic Research Center, Tehran university of Medical Sciences, Iran and also Ethics Committee for research on human subjects, Seth GsMedical College & KEM Hospital, Mumbai, India However the following issues were considered:

We have interviewed with patients who were agreed to participate. The consent was written only in KEM hospital and verbal in other centers. Identify was not revealed. The data was kept strictly confidential. Acknowledgment each particular centers when data published.

## Results

A total 717 individuals were included (381 of them were cases) mean age, weight and height of cases and controls broken down by the countries are shown in table 1. There was a good overall match between age range in two populations Mean of weight and BMI were significantly lower in osteoporotic group in both countries and mean of height was significantly lower in osteoporotic group in Indian subjects (p < 0.05) (table 1). Weight less than 60 kg and BMI less than 26 have been shown as risk factors of osteoporosis in both countries. Height less than 155 cm has been shown as a risk factor of osteoporosis in Indian subjects, see table 2.

**Table 2:** Distribution of associated factors with osteoporosis in two populations.

Variabels^1^	Iran				India			
	
	Case (n = 178)	Control (n = 185)	OR^2 ^(0.95 CI)	OR^3 ^(0.95 CI)	Case (n = 203)	Control (n = 151)	OR^2 ^(0.95 CI)	OR^3 ^(0.95 CI)
Education								
Subjects (n)	163	164	----	----	178	124	----	-----
<12 yrs schooling (%)	66.3	39.0	2.1 (1.7–4.3)	3.0 (1.8–5.1)	40.4	19.4	2.7 (1.5–4.8)	1.8 (0.8–4.0)
Anthropometric charcters (%)								
Weight <= 60 Kg (%)	37.0	12.9	3.9 (2.3–6.7)	----	54.9	20.0	4.8 (2.7–8.4)	-----
Height <= 155 Cm (%)	46.8	40.3	1.2 (0.8–1.8)	----	66.7	31.9	4.0 (2.3–7.2)	----
BMI <= 26 (%)	39.4	15.3	3.9 (2.3–6.7)	----	46.2	19.8	3.6 (1.9–6.6)	----
Menopausal factors								
Subjects (n)	162	183	----	----	159	105	----	----
Menopausal age before 45 yrs (%)	35.8	27.9	1.1 (1.04–1.1)	1.8 (1.0–3.0)	30.2	23.6	2.0 (1.2–3.5)	2.6 (1.2–5.9)
Subjects (n)	158	181	----	----	160	105	----	----
Postmenopausal >5 yrs (%)	75.3	52.5	2.2 (1.3–3.7)	2.3 (1.3–4.0)	73.5	58.5	1.4 (0.7–2.7)	1.4 (0.6–3.2)
Subjects (n)	149	143	----	----	79	64	----	----
Menarche after 14 yrs (%)	52.3	36.4	1.9 (1.1–1.3)	1.6 (0.9–2.6)	46.5	34.5	1.6 (0.8–3.4)	1.4 (0.5–3.9)
Subjects (n)	171	178	----	----	190	141	----	----
Parity >= 4 (%)	49.1	63.5	1.1 (1.0–1.1)	2.0 (1.2–3.3)	20.5	12.1	1.0 (1.04–1.1)	1.2 (0.5–2.6)
Medication								
Subjects (n)	178	179	----	----	183	138	----	----
Steroid usage (%)	19.1	7.3	3.3 (1.6–6.6)	3.3 (1.6–6.6)	5.5	1.4	4.3 (0.9–20.4)	NS^2^
Calcium supplementation>1 yr (%)	39.9	50.3	0.6 (0.4–0.9)	0.7 (0.4–1.0)	47.1	77.8	0.4 ((0.1–0.9)	0.2 (0.1–0.8)
HRT (%)	27.1	46.4	0.5 (0.3–0.7)	0.5 (0.3–0.7)	10.6	12.1	0.9 (0.4–1.8)	0.8 (0.3–2.4)
Other (%)								
Bone and join problem	29.4	15.1	2.3 (1.4–4.0)	2.5 (1.3–4.4)	35.7	20.7	2.2 (1.63–3.7)	1.8 (1.0–3.6)
History of fracture	20.1	8.5	2.6 (1.4–5.2)	3.3 (1.6–6.9)	20.7	7.1	3.2 (1.4–7.5)	3.8 (1.2–11.9)

1 = Variables are dichotomous. 2 = Non significant. OR2 = Adjusted for age. OR3 = Adjusted for age, height

Low education and husband low education and being housewife was shown as risk factors of osteoporosis in both populations, distribution of osteoporotic and controls based on education level is shown In table 2. Education less than 12 years of schooling remained significant (P < 0.05) as risk factor after age, height and weight adjustment in both countries.

Early menopause (before 45 years old), late menarche (after 14 years) and post menopausal duration more than 5 years were shown as significant risk factors in both countries. Parity more than 3 was shown as risk factor in both populations and it remained significant (P < 0.05) after age, height and weight adjustment in Iranian subjects (table 2).

According to table 2, bone and joint disorders and history of fracture during last 5 years were shown as risk factors of osteoporosis in both populations. Prevalence of osteoporosis was higher in women with history of osteoporosis and or fracture in their mother and sisters in both countries.

Hormone replacement therapy (HRT) and calcium supplementation in Iran and calcium supplementation more than 1 year in India have been shown as protective factors and medication with steroids was a risk factor for osteoporosis (Table 2).

Pure vegetarianism and Red meat consumption more than 4 times per week were shown as risk factors in Indian and Iranian subjects respectively. Regular consumption of Soya, almond fish, fruits and milk tea 4 cups per day and more appeared to be significant protective factors in India. Regular consumption of cheese, milk, chicken, egg, fruit, tea 7 cups per day and more were found to be significant protective factors in Iran. Distribution of subjects based on food consumption in both countries with their odds ratios are shown in table 3. In this study exercises was shown as protective factor in both countries and it remained significant after adjustment for age, weight and height in Iran. Walking and other exercises (aerobic weight lifting, swimming and other) were shown as protective factors in Iranian subjects (table 4).

**Table 3:** Distribution of nutritional associated factors with osteoporosis in two populations

Nutritional factors ^1^	Iran				India			
	
	Case (n = 178)	Control (n = 185)	OR^2 ^(0.95 CI)	OR^3 ^(0.95 CI)	Case (n = 203)	Control (n = 151)	OR^2 ^(0.95 CI)	OR^3 ^(0.95 CI)
Fish								
Subjects (n)	151	160	----	----	144	115	----	-----
>= 2/w (%)	4	6.9	NS	NS	6.9	21.7	0.5 (0.3–0.9)	0.4 (0.2–0.9)
Eggs								
Subjects (n)	135	148	----	----	146	115	----	----
>= 1/w (%)	55.6	68.9	0.6 (0.4–1.0)	0.6 (0.3–0.9)	28.1	42.6	0.5 (0.3–0.9)	0.6 (0.3–0.9)
Chicken								
Subjects (n)	169	165	----	----	196	145	----	----
>= 2/w (%)	58.6	74.5	0.4 (0.2–0.6)	0.4 (0.2–0.7)	16.7	17.1	NS^2^	NS
Red meat								
Subjects (n)	169	165	----	----	140	116	----	----
>= 4/w (%)	31.4	20	1.9(1.2–3.3)	1.9(1.1–3.3)	5	3.4	NS	NS
Almond								
Subjects (n)	149	143	----	----	79	64	----	----
>= 4/w (%)	13.9	16.5	NS	NS	17.7	32.1	0.5 (0.3–0.9)	0.4 (0.2–0.9)
Soya								
Subjects (n)	135	148	----	----	146	115	----	----
>= 4 (%)	1.2	4.3	NS	NS	7.2	19.6	0.3 (0.2–0.7)	0.4 (0.2–0.9)
Fruits								
Subjects (n)	166	164	----	----	131	101	----	----
Daily (%)	72.3	84.8	0.5 (0.3–0.9)	0.5 (0.3–0.9)	74	89.1	0.4 (0.2–0.8)	0.3 (0.1–0.7)
Tea (Iran)§/milktea (India)†								
Subjects (n)	172	170	----	----	163	113	----	----
>= 7 cup/w§and >= 4 cup/w† (%)	9.9	28.2	0.3 (0.2–0.6)	0.3 (0.1–0.5)	16.1	32.7	0.4 (0.2–0.8)	0.4 (0.2–0.9)
Milk								
Subjects (n)	176	173	----	----	135	112	----	----
>= 1 cup/day	51.1	63.0	0.5 (0.4–0.9)	0.6 (0.3–0.9)	62.2	62.5	NS^2^	NS^2^
Cheese								
Subjects (n)	167	169	----	----	122	108	----	----
>= 30 g/day	44.9	59.8	0.5 (0.4–0.8)	0.5 (0.3–0.9)	11.5	13.9	NS^2^	NS^2^

^1 ^– Variables are dichotomous.^2 ^– Non significant. OR2 = Adjusted for age. OR3 = Adjusted for age, height and weight

**Table 4:** Association of osteoporosis with exercises as protective factors in Iran and India.

Variabels^1^	Iran				India			
	
	Case (n = 178)	Control (n = 185)	OR^2 ^(0.95 CI)	OR^3 ^(0.95 CI)	Case (n = 203)	Control (n = 151)	OR^2 ^(0.95 CI)	OR^3 ^(0.95 CI)
Exercises								
Subjects (n)	178	185	----	----	202	149	----	-----
Yes (%)	77.0	90.8	0.4 (0.2–0.7)	0.4 (0.2–0.7)	87.2	78.1	0.4 (0.3–0.9)	NS^2^
Other exercises								
Subjects (n)	169	165	----	----	146	115	----	----
Yes (%)	21.9	44.8	0.4 (0.2–0.6)	0.3 (0.2–0.6)	16.0	17.8	NS^2^	NS^2^
Regular walking								
Subjects (n)	172	168	----	----	144	113	----	----
Yes (%)	68.6	84.5	0.5 (0.3–0.8)	0.4 (0.2–0.8)	72.3	77.4	NS^2^	NS^2^

^1 ^– Variables are dichotomous.^2 ^– Non significant. OR2 = Adjusted for age. OR3 = Adjusted for age, height and weight

The percentage of women who were directly in sunshine exposure at least for 15 minutes per day was significantly higher among controls compared to osteoporotic groups in Iranian subjects (P < 0.01). and sunshine exposure was shown as a protective factor in Iran Odds ratio and 95% confidence interval include 0.45 (0.28–0.72) and it remained significant after age, weight and height adjustment.

In this study frequency of smoking at least one per day in Iranian subjects were 9% (6% in osteoporotic group and 12% in controls), in Indian subjects it was %2.6 in total. The effective amount of cigarette smoking on osteoporosis is at least 10 cigarettes per day and its frequency were 3.6% and 1.4% respectively in Iran and India. More details of foundlings are available in tables S5 to S8 and charts 1 and 2 (see additional file 1).

## Discussions

In this study osteoporosis in Iranian and Indian subjects appears to be associated with several known risk factors that well described in the literature.

Aging is a major factor that affect bone mass and it was explained in several previous studies [29,30] in this study we have tried to control the effect of age on the study results by sampling cases and controls in similar age groups. But this kind of matching could not control the effect of age completely thus we have adjusted the result for age for tight control the effect.

### Demographic factors

Results of this study show that the education level is one of the most important demographic factors that associate with Osteoporosis. Reverse effects of education level on osteoporosis have been reported in some other studies [23,24].

The reason probably is the effect of education on lifestyle, nutrition and economic status. The other possibility is the effect of economic status in education level. People from well to do families have more facilities for continuing their education and they also have better nutritional and health status during childhood which affect the bone mass. Illiteracy and poverty are two main problems in heath preventive programs in Iran and India that should mentioned in future governmental strategies.

### Menstrual and obstetrical factors

Primary osteoporosis results from estrogen deprivation and constitutes 95% of all cases. The mechanism of estrogen deprivation in bone mass is well describe in previous studies [25-30]. Exposing by estrogen in longer time is associated with lower risk of osteoporosis. Thus menstrual factors such as late menarche age, early menopause, and amenorrhea have been shown as risk factors of osteoporosis in previous studies [31,32]. In this study Menstrual factors such as late menarche (after 14 years old), early menopause (before 45 years old) and Postmenopausal period more than 5 years has been shown as risk factors of osteoporosis in both countries. In present study it was also indicated that multi-parity more than 3 was a risk factor for osteoporosis in both countries. On balance of the available literature, it appears that there is a small loss of bone throughout pregnancy, between 1 and 4%, in the pelvis and lumbar spine. There is less than universal agreement about the forearm, but a smaller falls seems likely [33]. As mentioned above, biochemical indices of bone turnover are increased in the third trimester, and more so in women with multiple gestation.

According to birth rate report in 2005, 16.83 births per 1000 population in Iran and 22.32 births per 1000 population in India were reported [34]. The fertility rate was 1.82 and 2.78 children per woman in Iran and India respectively. It seems that with improving family planning program and decreasing fertility rate, multiparity may not be a risk factor of osteoporosis in next generation in two countries.

### Medical disorders and medication

Bone and joint problems (include any bone and joint discomfort that need treatment) have been shown as a risk factor of osteoporosis in Iranian and Indian subjects. The reason is probably effect of some diseases like rheumatoid arthritis, drugs like steroids and lack of ability for physical activity on osteoporosis.

#### Calcium supplementation

Prospective data show that calcium stabilized bone [35]. To prevent negative calcium balance, premenopausal women require 1000 mg and postmenopausal women 1200 mg of total elemental calcium daily [36]. In this study use of calcium supplements has been shown as a protective factor for osteoporosis in Iran. Use of calcium supplements more than 1 year has been shown as a protective factor in India.

#### HRT and Steroids

Estrogen deficiency after menopause predictably leads to bone loss and osteoporosis. Estrogen inhibits bone resorption produces a small rise in bone density, and reduces the risk of fracture by approximately 50 percent [37-39]. Accordingly, HRT is the accepted standard of practice for the prevention and for the treatment of osteoporosis. HRT and medication with steroids In Iran have been shown as protective and risk factor of osteoporosis respectively. In India percentage of women that were under HRT were higher in control group and percentage of women that use steroids were higher in osteoporotic group but these were not significant statistically. The probable reasons include:

a) Small number of women that were under HRT or steroid treatment in Indian subjects.

b) Use of HRT as a treatment after osteoporosis.

### Nutritional factors

The role of nutrition is perhaps the most controversial area in the causation of Osteoporosis. Calcium, phosphate, and vitamin D are essential for normal bone structure and function, but several other micronutrients also have essential roles in bone mass. Non nutrients such as phytoestrogens may also improve the status of bone tissue [40], Guthrie reports that women whose diets meet their calcium recommendation consume significantly more servings of milk and milk products and more several essential nutrients than women whose diet do not meet their calcium needs [41]. In India, nil consumption of milk, have been shown as risk factors of osteoporosis, In Iran, daily consumption of milk and cheese =>30 g/d have been shown as protective factors of osteoporosis. Also the consumption of almond, Soya products, were shown as protective factors in India [42]. Almond was also reported as good sources of calcium in literature. The isoflavones in soybeans, which function both as phytoestrogens and antioxidants, may result in the inhibition of bone resorption [43,44].

Vegetarian diets may be more beneficial than animal protein diet in many respects. But they may also contribute to a lower life time exposure to estrogen, which could increase the risk of osteoporotic fractures in vulnerable individuals, which could increase the risk of osteoporotic fractures in vulnerable individuals [45]. Some studies confirmed that there is no difference in bone health indices between lacto-ovo-vegetarians and omnivores and some of the studies propounded vegetarian diet as a risk factor for osteoporosis [46]. In this study pure vegetarianism has been shown as a risk factor of osteoporosis in India, although it was not significant after weight and height adjustment.

Present study also indicated that red meat consumption more than 4 times per week was a risk factor among Iranian subjects unlike chicken consumption more than 2 times per week that was shown as a protective factor. Excessive protein consumption may lead to increased urinary calcium excretion. Although high calcium intakes are not significantly affected by a high protein intake, low calcium intakes are generally not sufficient to offset a high protein intake. Also important is total protein in the diet. Low levels of serum albumin negatively affect transport of serum calcium. Fracture patients may be especially vulnerable to the relationship between low calcium and high protein intakes [46].

Protective role of chicken, fish and egg at present study in Iran confirm the theory of "adequate protein intake is important for optimal bone health in the elderly 50–69 years of age". But red meat consumption 4 times or more per week was shown as a risk factor in Iranian population. The most probably reasons are as follow:

1 – People who use fish and chicken more than 2 times per week are those who are following medical healthy diet with balance in red meat and white meat consumption. Thus their dietary pattern is not associated with excessive consumption of protein.

2 – Red meat and white meat probably have different biological effects on bone mass. For example there is higher proportion of phosphorous in red meat that may affect bone mass [46]. However this theory needs to be confirmed with other studies.

According to previous studies, higher fruit and vegetable intake was associated with greater BMD in men and women [47,48]. In this study daily consumption of fruits was shown as a protective factor in Iranian and Indian subjects. There was no significant association with vegetable consumption and osteoporosis in Iran and India.

In this study black tea consumption more than 6 cups per day in Iran and Milk tea consumption 4 cups per day or more in India have been shown as protective factors for osteoporosis. Similar results have been reported in some previous studies [49]. Nutrients found in tea, such as flavonoids, may influence BMD [50].

### Physical activity and Exercise

Exercises have been shown as a protective factor in both countries. Recent evidence indicates that some forms of physical activity may maintain or even increase BMD in selected population [51,52]. Weight bearing exercise and muscle contraction combined have been shown to effectively increase bone density in the spine. It is recommended that an individual perform 20 to 30 minutes of aerobic exercise 3 to 4 times weekly to increase bone mass. Simple exercises such as walking can help strengthen bones and muscles there is strong evidence that physical activity begun early in life contributes to higher peak bone mass [19,53]. In this study women with no regular walking were in more risk of osteoporosis in Iran. There was no significant protective role in walking or the time and duration of walking in Indian subjects. Other kinds of exercises like aerobic, swimming, weight lifting and others have been shown as a protective factor in Iran.

### Anthropometric Factors

High weight is a protective factor for osteoporosis, in obese individuals; fracture risk is reduced [40]. In this study weight less than 60 kg and BMI less than 26 have been shown as risk factors of osteoporosis in both countries. Height less than 155 cm have been shown as a risk factor of osteoporosis in Indian subjects and in private center of Iran. The role of anthropometric factors was reported in previous studies [54].

### Habits

Cigarette smoking is a risk factor for vertebral, forearm and hip fractures. Women who smoke enter menopause 1 to 2 years earlier and lose bone more rapidly than non smokers [55]. Women who smoke about one pack of cigarettes daily will have an average deficit of 5% to 10% in bone density which increase the risk of fracture [40] in this study Frequency of smoking at least one per day in Iranian subjects were 9% (6% in osteoporotic group and 12% in controls), in Indian subjects it was 2.6% in total. The effective amount of cigarette smoking on osteoporosis is at least 10 cigarettes per day and its frequency were 3.6% and 1.4% respectively in Iran and India thus it seems because of small amount of smoking per day it does not show any effect on osteoporosis in both populations.

### Fracture history

the major problem related to osteoporosis is fracture and the significant association between history of fracture with low bon density and osteoporosis are well described in previous studies [30,54] in present study also we have found the same results in both countries.

## Conclusion

Osteoporosis in Iranian and Indian subjects also appears to be associated with several known risk factors that well described in the literature. There were no significant differences in association of risk factors and osteoporosis between Iranian and Indian subjects.

It was shown a protective role of certain nutritional dietary components and also exercises in both populations and can be exploited in preventive educational strategies on osteoporosis in these populations

## Competing interests

The author(s) declare that they have no competing interests.

## Authors' contributions

A. Keramat contributed in study design and acquisition of data and analysis B. Patwardhan and A. Chopra, contributed in study design and supervision and guidance during study period and also supervision in making the final report, B Larijani A. contributed in study design and supervision during the study period in Iran, Mithal, D., Chakravarty D; contributed in study design and supervision during the study period in New Delhi. H Adibi: acquisition of data, involved in drafting the manuscript. A Khosravi rechecked statistical analysis and involved in edit the manuscript. All authors approved the manuscript.

## Pre-publication history

The pre-publication history for this paper can be accessed here:



## Supplementary Material

Additional file 1 details. Includes the most important descriptive information Click here for file
