# Supplementary material for: The assessment of osteoporosis risk factors in Iranian women compared with Indian women
Source: BMC Musculoskelet Disord. 2008 Feb 27;9:28. doi: 10.1186/1471-2474-9-28 (PMC2289820; doi:10.1186/1471-2474-9-28)
Supplement: Additional file 1 — details. Includes the most important descriptive information [file 1471-2474-9-28-S1.doc]

Table () Number and mean age of osteoporotic and controls in Iran and India

| **Diagnosis** | | **osteoporosis** | | | **normal** | | | **Total** | | |
| --- | --- | --- | --- | --- | --- | --- | --- | --- | --- | --- |
| **Country** | **No.** | | **Mean age** | **SD** | **No.** | **Mean age** | **SD** | **No.** | **Mean age** | **SD** |
| **Iran** | **178** | | **58.2** | **7.1** | **185** | **55.7** | **6** | **363** | **56.9** | **6.7** |
| **India** | **203** | | **58.9** | **8.1** | **151** | **56.4** | **7.5** | **354** | **57.9** | **8** |
| **both country** | **381** | | **58.6** | **7.7** | **336** | **56** | **6.7** | **717** | **57.4** | **7.4** |

Table () Mean of menopausal age based of osteoporotic and controls in Iran and India:

|  | Country | Iran | | | India | | |
| --- | --- | --- | --- | --- | --- | --- | --- |
| Menopausal age* | diagnosis | N | Mean | SD | N | Mean | SD |
|  | osteoporotic | 162 | 46.55 | 5.84 | 159 | 47.09 | 6.08 |
|  | controls | 183 | 47.84 | 5.06 | 105 | 48.20 | 4.33 |
|  | Total | 345 | 47.23 | 5.47 | 264 | 47.53 | 5.47 |
| Post Menopausal duration* | osteoporotic | 158 | 11.60 | 7.42 | 160 | 12.01 | 8.64 |
|  | controls | 181 | 7.72 | 6.21 | 105 | 8.24 | 6.55 |
|  | Total | 339 | 9.53 | 7.06 | 265 | 10.52 | 8.08 |
| Menarche age* | osteoporotic | 149 | 13.78 | 1.70 | 79 | 13.50 | 1.56 |
|  | controls | 143 | 13.24 | 1.56 | 64 | 12.98 | 1.33 |
|  | Total | 292 | 13.52 | 1.65 | 143 | 13.27 | 1.48 |

* Significant differences between osteoporotic and controls in Iran and India (P<0.05)

Table () Mean of menopausal age based of osteoporotic and controls in Iran and India:

|  | Country | Iran | | | India | | |
| --- | --- | --- | --- | --- | --- | --- | --- |
| Menopausal age* | diagnosis | N | Mean | SD | N | Mean | SD |
|  | osteoporotic | 162 | 46.55 | 5.84 | 159 | 47.09 | 6.08 |
|  | controls | 183 | 47.84 | 5.06 | 105 | 48.20 | 4.33 |
|  | Total | 345 | 47.23 | 5.47 | 264 | 47.53 | 5.47 |
| Post Menopausal duration* | osteoporotic | 158 | 11.60 | 7.42 | 160 | 12.01 | 8.64 |
|  | controls | 181 | 7.72 | 6.21 | 105 | 8.24 | 6.55 |
|  | Total | 339 | 9.53 | 7.06 | 265 | 10.52 | 8.08 |
| Menarche age* | osteoporotic | 149 | 13.78 | 1.70 | 79 | 13.50 | 1.56 |
|  | controls | 143 | 13.24 | 1.56 | 64 | 12.98 | 1.33 |
|  | Total | 292 | 13.52 | 1.65 | 143 | 13.27 | 1.48 |

* Significant differences between osteoporotic and controls in Iran and India using T test(P<0.05)

Table () Distribution of subjects among osteoporotic and controls based on

education level in Iran and India

| **Education level** | **Iran (%)** | | **India (%)** | |
| --- | --- | --- | --- | --- |
| Years schooling | **Osteoporosis**  **n=163** | **Control n=164** | **Osteoporosis n=180** | **India n=126** |
| No | 19.6 | 6.7 | 9.4 | 4 |
| 1-5 | 32.5 | 18.9 | 7.8 | 4 |
| 6-8 | 2.5 | 3 | 12.8 | 2.5 |
| 9-11 | 4.5 | 10.4 | 11.1 | 9.5 |
| >12 | 33.4 | 61 | 58.9 | 80.2 |
| **Husband education level** | **Osteoporosis n=163** | **Control n=164** | **Osteoporosis (% n=180** | **Control n=126** |
| No | 12.6 | 2.8 | 8.6 | 3.2 |
| 1-5 | 24.4 | 11.9 | 3.7 | 3.2 |
| 6-8 | 5 | 1.8 | 8.6 | 4.8 |
| 9-11 | 4.2 | 6.4 | 8.6 | 6.5 |
| >12 | 53.8 | 77.1 | 70.5 | 62.3 |
